# IgTM: An algorithm to predict transmembrane domains and topology in proteins

**DOI:** 10.1186/1471-2105-9-367

**Published:** 2008-09-10

**Authors:** Piedachu Peris, Damián López, Marcelino Campos

**Affiliations:** 1 Departamento de Sistemas Informáticos y Computación. Universidad Politécnica de Valencia. Camí de Vera s/n 46071 Valencia. Spain

## Abstract

**Background:**

Due to their role of receptors or transporters, membrane proteins play a key role in many important biological functions. In our work we used Grammatical Inference (GI) to localize transmembrane segments. Our GI process is based specifically on the inference of Even Linear Languages.

**Results:**

We obtained values close to 80% in both specificity and sensitivity. Six datasets have been used for the experiments, considering different encodings for the input sequences. An encoding that includes the topology changes in the sequence (from inside and outside the membrane to it and vice versa) allowed us to obtain the best results. This software is publicly available at:

**Conclusion:**

We compared our results with other well-known methods, that obtain a slightly better precision. However, this work shows that it is possible to apply Grammatical Inference techniques in an effective way to bioinformatics problems.

## Background

Membrane proteins are involved in a variety of important biological functions [1,2] where they play the role of receptors or transporters. The number of transmembrane segments of a protein and some characteristics such as loop lengths can identify features of the proteins, as well as their role [3]. Therefore, it is very important to predict the location of transmembrane domains along the sequence, since these are the basic structural building blocks defining the protein topology. Several works have dealt with this prediction task from different approaches, mainly using Hidden Markov Models (HMM) [4-6], neural networks [7,8] or statistical analysis [9]. A rich literature is available on proteins prediction. For reviews on different methods for predicting transmembrane domains in proteins, we refer the reader to [10-12].

**Figure 1:**
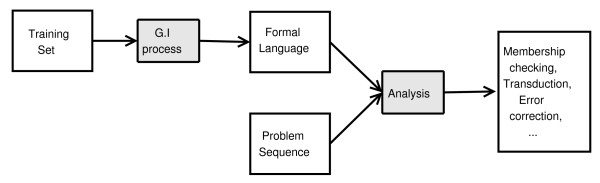
**Process of a Grammatical Inference process**. A formal language can be represented by means of an automaton or a grammar, that will be used together with the problem sequences in the analysis phase. The output of the analysis phase can be a transduction of the input sequence, an error-free form of the input, or a value that tells whether the sequence belongs to the language or not.

This work addresses the problem of protein transmembrane domains prediction by making use of a Grammatical Inference (GI) based approach. GI is a particular case of Inductive Inference, an iterative process that takes into account a set of facts and tries to obtain a model consistent with the available data. In GI the model resulting from the induction process is a formal grammar (that generates a formal language) inferred from a set of sample strings, composed by a set *M*^+ ^of strings belonging to a target formal language and, in some cases, another set *M*^- ^of strings that do not belong to the language. The results of the inference process gives as the result a language (hypothesis) that, in essence, models all the common features of the strings. This grammatical approach is suitable for the task due to the sequential nature of the information. Some works apply formal languages methods to molecular biology [13]. Figure 1 depicts a general GI scheme. Several classifications of the GI algorithms can be made, for instance: when both sets are non-empty we remalgo[cont2]Algorithm refer to complete presentation algorithms; positive presentation algorithms are those that use an empty *M*^- ^set; taking into account these algebraic properties of the obtained languages, it is possible to distinguish between characterisable and non-characterisable algorithms. It is difficult to identify what information is suitable to be considered into *M*^-^, therefore we will take into account only positive presentation in our approach. For more information, we refer the reader to [14-16].

Usually, the model used in GI is a finite state abstract machine commonly named finite automata. HMMs are closely related to finite automata, and therefore our approach is also related to several works that succesfully tackle this task [4-6]. Nevertheless, it is to note that the topology of a HMM, number of states and their connection, is a priori fixed by an expert that takes profit from known information. Once the topology is fixed, the available data is used to set the probability of each transition of the HMM. As stated above, the input of a GI algorithm is a set of sequences, therefore no aid from an expert is needed, because both the topology of the automaton and the probability between states is automatically stablished by the algorithm.

Generally speaking, HMMs provide a good solution when the topology of the HMM can be fairly set. In that case, the sequences provided are used just to set the transition probabilities among states. A GI approach tries to extract more information from the sequences and provides good prediction tools using only sequential information. The most important drawback of GI is the lack of enough data to infer proper models.

GI has been used previously in various bioinformatics related tasks, such as gene-finding or prediction of *coiled coil *domains [17]. The good performance of those works leds us to apply GI algorithms to the prediction of other domains in proteins, such as transmembrane segments.

Our work takes into account a set of protein sequences with known evidence of transmenbrane domains. Firstly, these sequences are processed in order to distinguish among inner, outer and transmembrane residues. This labelling allows to obtain an *Even Linear structure *(that considers a relationship among the symbols in a sequence, such that the first and the last symbols are related, the second and the last but one are also related and so on). It is possible to model this structure by using an Even Linear Language (ELL) that can be learned using GI techniques. The obtained language is then used to build a probabilistic transducer (an abstract machine that processes an input sequence and obtains another output sequence or transduction with an occurrence probability). The resulting transducer allows to process any unknown protein sequence to obtain a transduction. The transduction shows those detected transmembrane domains. The experimental results have been compared with TMHMM 2.0 [4], Pred-TMR [9], Prodiv-TMHMM [6], HMMTOP 2.0 [18,5], PHOBIUS [19-21], TMpred [22,23] and MEMSAT3 [24].

## Results and discussion

### Introduction

We consider the prediction of transmembrane domains as a transduction problem. That is, given an amino acid sequence, the output of our system is a sequence with the same length which distinguishes between those amino acids that are within a transmembrane domain and those that are not.

The available data are transformed in a training set with even linear structure. An item of the data set is a string whose first half is made up by the symbol sequence of the protein and the second by the symbols of the expected output string in reverse order. In order to learn the ELL with this set, we considered as the main feature the segments of a given length *k *set as a input parameter. The class of *k*tss languages is a well-known subclass of the regular languages and it is characterized by the set of segments of length *k *that appear in the words of the language, therefore, we can take profit of previous learning results in order to address this task [25-27].

The transducer is obtained using the structure of the inferred ELG (Even Linear Grammar). The general method is described in Algorithm 1. Please refer to section *Notation and definition *to details.

### Algorithm 1 Transmembrane Grammatical Inference approach

*Input:*

*• A set P of amino acid sequences with known transmembrane domains*.

*• A set L of domain labeled sequences. Each string x in P has its corresponding string l_x _in L*.

*Output:*

*• A transducer to locate transmembrane domains*.

*Method:*

• Combine the sets P and L to obtain the training set M with strings $xl_{x}^{r}$

• Apply to the strings in M the transformation function σ

• Apply a GI algorithm for (a subclass of) regular languages

• Undo the transformation σ to obtain the ELG from the regular language

• Return the transducer obtained from the ELG

*End*

The returned transducer can be used to analyse problem sequences to obtain the corresponding transduction.

### Datasets

Due to the fact that each approach to transmembrane prediction uses its own dataset, in order to test our approach six different datasets has been considered. The first one was a set of 160 membrane proteins used in [4], which we refer to as the TMHMM set. Experimental topology data is available for these proteins, most of them have been analysed with biochemical and genetic methods (these methods are not always reliable), and only a small number of membrane protein domains of this dataset have been determined at an atomic resolution. The dataset contains 108 multi-spanning and 52 single-spanning proteins. The original dataset was larger, but those proteins whith conflicting topologies for different experiments were not included.

The second set used was TMPDB [28], whose latest version (Release 6.3) contains 302 transmembrane protein sequences (276 alpha-helical sequences, 17 beta-stranded sequences and 9 alpha-helical sequences with short pore-forming alpha-helices buried in the membrane). The topologies of these sequences are based on definite experimental evidences such as X-ray crystallography, NMR, gene fusion technique, substituted cysteine accessibility method, Asp(N)-linked glycosylation experiment and other biochemical methods. The third and fourth datasets are subsets of TMPDB, where homologous proteins have been removed: the third set, TMPDB-*α*-nR, contains 230 alpha-helix non redundant proteins; and the fourth set TMPDB-*αβ*-nR, has been obtained by adding 15 *β*-barrel proteins to the third set.

The fifth dataset used is the 101-Pred-TMR database, a set of 101 non-homologous proteins, extracted form SwissProt database, used in [9,29]. These proteins were selected from a set of 155 proteins, discarding those with more than 25% of similarity.

The last dataset used was the MPTOPO dataset [30]. In its last version (August 2007) the set contains 185 proteins: 25 of them *β*-barrels and the rest *α*-helix transmembrane. All the segments have been experimentally validated. The 3D structure of 119 of these proteins has been determined using x-ray diffraction or NMR methods, therefore, these transmembrane segments are known precisely. The rest of transmembrane segments correspond to 41 helices that have been identified by experimental techniques such as gene fusion, proteolytic degradation, and amino acid deletion. The proteins whose topologies are based solely on hydropathy plots have not been included in the dataset.

### Codification

Protein sequences can be considered as strings from a 20 symbols alphabet, where each symbol represents one of the amino acids. In order to reduce the alphabet size without loss of information, we considered an encoding based on some properties of the amino acids (originally proposed by Dayhoff). The Table 1 shows the correspondence of each amino acid for Dayhoff encoding. This encoding has been previously used in some GI papers [31-33].

**Table 1:** Amino acid encoding.

Amino acid	Property	Dayhoff
C	sulfur polymerization	a
G, S, T, A, P	small	b
D, E, N, Q	acid and amide	c
R, H, K,	basic	d
L, V, M, I	hydrofobic	e
Y, F, W	aromaticity	f

The Dayhoff encoding takes into account the properties of each residue: sulfur polymerization, small, acid and amide, basic, hydrophobic and aromaticity. For example, for the input protein segment SVMEDTLLSVLFETYNPKVRPAQTVGDKVTVRV the output encoded sequence would be: beeeccbeebeefcbfcbdedbbcbebcdebede

### Performance measures

Several measures are suitable to evaluate the results. Some of them, addressing gene-finding problems, are reviewed in [34]. This measures can also be applied to functional domain location tasks. Among all the proposed measures, *Sensitivity *and *Specificity *are probably the most used. Intuitively, Sensitivity (*Sn*) measures the probability of predicting a particular residue inside a domain. Specificity (*Sp*) measures the probability of predicted residues to be actually into a domain. Therefore, *Sn *and *Sp *can be computed as follows:

$$\begin{array}{cc} Sn=\frac{TP}{TP+FN} & Sp=\frac{TP}{TP+FP} \end{array}$$

Where:

**True positives (TP): **correctly localized amino acids into a TM domain.

**True Negatives (TN): **correctly annotated amino acids out of a TM domain.

**False positives (FP): **amino acids out of a TM domain annotated as belonging to a domain.

**False Negative (FN): **amino acids into a TM domain not correctly localized (annotated as out of any domain).

Note that neither *Sn *nor *Sp*, took individually, constitute an exhaustive measure. A single value that summarizes both measures into a better one is the *Correlation Coefficient *(*CC*), also referred to as Mathews Correlation Coefficient [35]. It can be computed as follows:

$$CC=\frac{\left(TP\cdot TN)-(FN\cdot FP\right)}{\sqrt{\left(TP+FN)\cdot (TN+FP)\cdot (TP+FP)\cdot (TN+FN\right)}}$$

Unfortunately, although *CC *has some interesting statistical properties [34], it has also an undesirable drawback. It is not defined if any factor of the root is equal to zero. In the literature there exist some measures that overcome this inconvenient, in this work we will use the *Approximate Correlation *(*AC*) which is defined as follows:

$$\begin{array}{c} ACP=\frac{1}{4}\left[\frac{TP}{TP+FN}+\frac{TP}{TP+FP}+\frac{TN}{TN+FP}+\frac{TN}{TN+FN}\right]\\ AC=(ACP-0.5)\cdot 2 \end{array}$$

We have to note that we were not able to calculate *CC *for every sample of the testing set (independently the dataset considered). In those cases, the samples were not taken into account. The Approximate Correlation *AC *has a 100% coverage, including those samples for which it was not possible to calculate *CC *or *Sp*. This can explain the relevant difference between *AC *and *CC *observed in some experiments. In addition to this, we have used the common segment-based measure Segment overlap, (*Sov*_*δ*_^*obs*^) defined by [36]:

$$Sov_{\delta }^{obs}=\frac{1}{N}\sum_{s}\frac{min(E)-max(B)+1+\delta }{max(E)-min(B)+1}len(s_{1})$$

where *N *is the total number of residues observed within all the domains of the protein, *s*_1 _and *s*_2 _are two overlaped segments, *E *is {*end*(*s*_1_); *end*(*s*_2_)}, *B *is {*beg*(*s*_1_); *beg*(*s*_2_)} and *δ *is a parameter for the accepted (maximal) deviation. We used a value of *δ *= 3.

**Figure 2:**
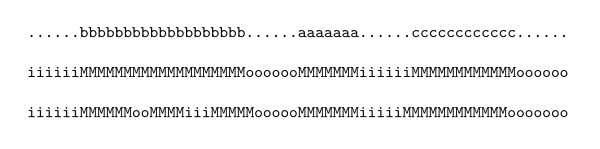
**Example of segments correctly predicted with different levels of precision. **The first line shows the protein to predict, the second one is the prediction. This example shows a segment completely predicted (a), one segment correctly predicted at least at 75\% (b) and one segment predicted at least at 90\% (c).

We have also calculated the number of segments correctly predicted at three accuracy thresholds: 100%, 90% and 75%, that is, number of segments with the 100%, 90% or more, and 75% or more of their amino acids are correctly predicted. This measure is similar to Sensibility, but it is based on segments. Therefore it is necessary to calculate also the Sp measure in order to complement it. This measure allows to obtain a reliable evaluation for those segments that contain false negatives not only at the extremities of the segment. For example, this occurs when a viewed segment is recognized as more than one segment, and there are some false negatives between two of this predicted segments. Figure 2 shows how this measure is calculated.

### Experimentation

Note that our approach needs some information to learn a model. In order to obtain probabilistic relevance in the test of our method, we followed a *leaving one out *scheme: each sample protein of the dataset is annotated using as training set all the other samples. The process is repeated until all sample proteins have been used as test sequences. We carried out various experiments, taking into account different annotations for the test sequences. Each experiment was carried out over the six databases TMHMM, TMPDB, TMPDB-*α*-nR, TMPDB-*αβ*-nR, 101pred-tmr and MPTOPO. Note that all these sets but TMPDB have non homologous sequences.

We hereby provide a description of each experiment, all the experiments but the last consider a previous reduction using the Dayhoff code: The first one (*exp1*) considered a two-classes encoding, that is, residues inside and outside a transmembrane domain; the second experiment (*exp2*) added another class in order to consider the topology of the protein (inner and outer residues); the third experiment (*exp3*) also included a class to distinguish among transmembrane domains with previous inner and outer regions; the fourth (*exp4*) experiment took into account the previous encoding with a special labelling of the last five residues of each region preceding a transmembrane one; the fifth experiment (*exp5*) added special symbols to track the transition to a transmembrane and out to one; the last experiment (*exp6*) did not consider the Dayhoff encoding and used the annotation of the second experiment.

**Figure 3:**
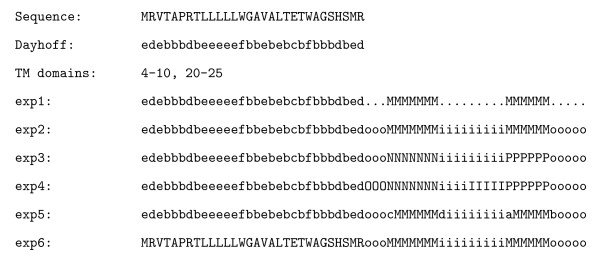
**Examples of sequences annotated and codified for each experiment. **This figure shows the annotation  and encoding  of an example sequence for each different experimental configuration.

Each of the experiments builds a different model for the language of the TM proteins, that highlights differents propierties of them, by searching different patterns among the amino acids, depending on whether they belong, for instance, to a TM zone or not (*exp1*), to an inner or outer zone (*exp2 *and *exp6*), to a TM domain with previous inner or outer regions (*exp3*), to the sequence of the last 5 residues that precede a TM segment (*exp4*), or to the set of amino acids that represent a transition from a TM zone to an inner or outer zone, or vice versa (*exp5*). Figure 3 shows the annotation and encoding of an example sequence for each different experimental configuration.

Once encoded the sequences, and for each of the described encodings, a set of experiments were run to test the best learning parameter of the inference algorithm. The best accuracy was obtained in the experiment with the configuration of *exp*_5 _and *exp*_6_. The HMM-based methods we compared our system with, obtain a slightly better precision. The difference in results can be explained with the fact that GI algorithms need a greater quantity of data than the amount needed by Hidden Markov Models in order to achieve the same accuracy.

The main advantage of our approach is that it learns the topology of the model from samples, without the need of the external knowledge, as in HMM-based methods, where states and edges are determined by an expert. In a GI method, the automata are built by the algorithm, which stablishes the topology, number of states, the transitions or edges between states and probabilities of transition. Tables 2, 3, 4, 5, 6 show the experimental results of the fifth and sixth experiments (those which returned the best results) with the six datasets.

**Table 2:** Experimental results TMHMM.

		**TMHMM database**							
		
		**Sn**	**Sp**	**CC**	**AC**	*Sov*_3_^*obs*^	100%	≥ 90%	≥ 75%
**igTM**	**exp5**	0.808	0.810	0.707	0.702	0.680	0.474	0.603	0.756
	
	**exp6**	0.819	0.796	0.715	0.707	0.707	0.490	0.618	0.789

**TMHMM 2.0**		0.900	0.879	0.830	0.827	0.915	0.339	0.636	0.920
**Pred-TMR**		0.786	0.898	0.769	0.767	0.843	0.200	0.378	0.723
**Prodiv-TMHMM**		0.832	0.854	0.778	0.768	0.866	0.269	0.523	0.848
**HMMTOP**		0.849	0.894	0.810	0.802	0.913	0.255	0.537	0.844
**PHOBIUS**		0.891	0.850	0.804	0.924	0.936	0.367	0.635	0.902
**MEMSAT3**		0.896	0.843	0.799	0.912	0.801	0.412	0.750	0.926
**TMpred**		0.834	0.796	0.741	0.738	0.899	0.331	0.540	0.813

Experimental results and comparison with results of other methods, with the TMHMM *α*-helix database. As discussed in the Performance Measures section, 100%, ≤ 90% and ≥ 75% stand for segments correctly detected in that percentage.

**Table 3:** Experimental results TMPDB.

		**TMPDB**							
		
		**Sn**	**Sp**	**CC**	**AC**	*Sov*_3_^*obs*^	100%	≥ 90%	≥ 75%
**igTM**	**exp5**	0.683	0.750	0.587	0.539	0.519	0.444	0.515	0.608
	
	**exp6**	0.710	0.759	0.617	0.557	0.533	0.487	0.562	0.652

**TMHMM 2.0**		0.739	0.831	0.717	0.659	0.745	0.259	0.465	0.671
**Pred-TMR**		0.777	0.899	0.785	0.756	0.831	0.209	0.426	0.736
**Prodiv-TMHMM**		0.737	0.829	0.709	0.659	0.756	0.208	0.427	0.647
**HMMTOP**		0.769	0.802	0.686	0.861	0.670	0.189	0.381	0.651
**PHOBIUS**		0.775	0.786	0.686	0.670	0.811	0.258	0.452	0.693
**MEMSAT 3**		0.775	0.793	0.668	0.671	0.783	0.278	0.487	0.692
**TMpred**		0.702	0.755	0.615	0.598	0.746	0.222	0.361	0.572

Experimental results and comparison with results of other methods, with the TMPDB *α*-helix database. As discussed in the Performance Measures section, 100%, ≥ 90% and ≥ 75% stand for segments correctly detected in that percentage.

**Table 4:** Experimental results TMPDB-*α*-nR.

		**TMPDB-*α*-nR**							
		
		**Sn**	**Sp**	**CC**	**AC**	*Sov*_3_^*obs*^	100%	≥ 90%	≥ 75%
**igTM**	**exp5**	0.637	0.757	0.571	0.533	0.488	0.431	0.516	0.623
	
	**exp6**	0.698	0.771	0.618	0.578	0.511	0.487	0.575	0.674

**TMHMM 2.0**		0.814	0.813	0.728	0.710	0.818	0.335	0.569	0.805
**Pred-TMR**		0.775	0.826	0.696	0.700	0.823	0.183	0.376	0.688
**Prodiv-TMHMM**		0.823	0.802	0.720	0.712	0.841	0.272	0.543	0.802
**HMMTOP**		0.823	0.782	0.699	0.699	0.875	0.257	0.486	0.777
**PHOBIUS**		0.845	0.791	0.717	0.860	0.881	0.333	0.559	0.847
**MEMSAT 3**		0.820	0.808	0.711	0.714	0.828	0.328	0.582	0.808
**TMpred**		0.765	0.747	0.643	0.643	0.813	0.264	0.441	0.673

Experimental results and comparison with results of other methods, with the TMPDB-*α*-nR *α*-helix database. As discussed in the Performance Measures section, 100%, ≤ 90% and ≥ 75% stand for segments correctly detected in that percentage.

**Table 5:** Experimental results MPTOPO.

		**MPTOPO**							
		
		**Sn**	**Sp**	**CC**	**AC**	*Sov*_3_^*obs*^	100%	≥ 90%	≥ 75%
**igTM**	**exp5**	0.651	0.708	0.443	0.439	0.491	0.312	0.400	0.509
	
	**exp6**	0.672	0.752	0.511	0.469	0.576	0.409	0.472	0.572

**TMHMM 2.0**		0.634	0.882	0.663	0.563	0.666	0.121	0.240	0.465
**Pred-TMR**		0.572	0.893	0.617	0.542	0.630	0.061	0.145	0.345
**Prodiv-TMHMM**		0.609	0.887	0.643	0.547	0.647	0.087	0.199	0.410
**HMMTOP**		0.630	0.868	0.637	0.558	0.683	0.084	0.175	0.415
**PHOBIUS**		0.640	0.884	0.670	0.576	0.687	0.105	0.225	0.466
**MEMSAT 3**		0.667	0.821	0.581	0.584	0.701	0.139	0.283	0.506
**TMpred**		0.578	0.831	0.567	0.506	0.622	0.096	0.182	0.383

Experimental results and comparison with results of other methods, with the MPTOPO *α*-helix database. As discussed in the Performance Measures section, 100%, ≥ 90% and ≥ 75% stand for segments correctly detected in that percentage.

**Table 6:** Experimental results 101pred-tmr.

		**101-PRED-TMR-DB**							
		
		**Sn**	**Sp**	**CC**	**AC**	*Sov*_3_^*obs*^	100%	≥ 90%	≥ 75%
**igTM**	**exp5**	0.793	0.821	0.697	0.692	0.651	0.522	0.613	0.725
	
	**exp6**	0.801	0.820	0.718	0.709	0.714	0.423	0.577	0.739

**TMHMM 2.0**		0.899	0.871	0.822	0.817	0.909	0.346	0.625	0.899
**Pred-TMR**		0.814	0.909	0.792	0.795	0.873	0.229	0.416	0.751
**Prodiv-TMHMM**		0.831	0.840	0.772	0.760	0.864	0.297	0.542	0.828
**HMMTOP**		0.862	0.890	0.811	0.808	0.926	0.258	0.546	0.846
**PHOBIUS**		0.895	0.836	0.798	0.796	0.936	0.375	0.636	0.883
**MEMSAT 3**		0.889	0.834	0.787	0.790	0.893	0.432	0.732	0.913
**TMpred**		0.845	0.775	0.725	0.732	0.908	0.343	0.530	0.798

Experimental results and comparison with results of other methods, with the 101pred-tmr *α*-helix database. As discussed in the Performance Measures section, 100%, ≥ 90% and ≥ 75% stand for segments correctly detected in that percentage.

Although it may seem erroneous or non-sense to build a model to predict both *α *and *β *transmembrane domains, we would like to illustrate with this experiment the way a GI approach distinguishes from other approaches: if the dataset contains enough data (sequences in our case) from differents classes (*α*-helices and *β*-barrels), the model obtained should be able recognize all the different patterns. Table 7 compares the results of the experiment carried out over TMPDB-*α*-nR and TMPDB-*αβ*-nR datasets. The results with TMPDB-*αβ*-nR are slightly worse, but it can be explained because the set of *β*-barrel proteins contains only 15 sequences, and it is difficult to learn an accurate model from this set. In fact, when we train and test with only this set of *β*-barrel proteins (which would be TMPDB-*β*-nR) the result are roughly worse: (results from *exp5*) 0.506 for *Sp*, 0.170 for *AC *and 0.318 for *Sov*_3_^*obs*^; and in *exp6*:0.541 for *Sp*, 0.270 for *AC *and 0.584 for *Sov*_3_^*obs*^.

**Table 7:** igTM experimental comparison when TMPDB-*α*-nR and TMPDB-*αβ*-nR datasets were taken into account.

		**Sn**	**Sp**	**CC**	**AC**	*Sov*_3_^*obs*^	100%	≥ 90%	≥ 75%
**TMPDB-*αβ*-nR**	**exp5**	0.618	0.732	0.545	0.498	0.462	0.412	0.481	0.564
	
	**exp6**	0.676	0.750	0.600	0.542	0.476	0.471	0.547	0.621

**TMPDB-*α*-nR**	**exp5**	0.637	0.757	0.571	0.533	0.488	0.431	0.516	0.623
	
	**exp6**	0.698	0.771	0.618	0.578	0.511	0.487	0.575	0.674

## Conclusion

This work addresses the problem of the localization of transmembrane segments within proteins by making use of Grammatical Inference (GI) algorithms. GI has been effectively used in some bioinformatic related tasks, such as gene-finding or prediction of *coiled coil *domains. IgTM exploits the features of proteins by using Even Linear Languages as the inferred class of languages. We tested different labellings for the input sequences, with the best accuracy achieved using a labelling that takes into account several changes in the sequence topology: from inside and outside the membrane to it and vice versa. We compared our method with other methods to predict transmembrane domains in proteins, obtaining slightly less accuracy with respect to them. This should be due to the fact that in GI the training phase need more data than the most common approach, based on Hidden Markov Models. In addition to this, many of the available prediction tools are *closed*, that is, there is no way to know exactly the training set used by the tools which we have compared igTM with, therefore it is possible that some of our six datasets included proteins used by these tools in the training phase (in this case, the tools we compare our algorithm with, would obtain better results). The same problem happens with online prediction tools, where the data considered to build the tools is not available. Then, since the other methods can have been trained on sequences that share homology with the test set (or even sequences included in the test set), the comparison could be not very reliable. However, the obtained results show that GI can be used effectively in bioinformatics related tasks. Furthermore, the main advantage of GI when applied to bioinformatics tasks is that an expert is not needed in order to give additional information (in this case the topology of transmembrane proteins). An online version of IgTM is publicly available at 

It remains as a future work to use this method together with another one (based on HMM or not). This could lead to improve the performance. At present we are testing other inference algorithms to learn the automata, the use of new codings to the sequences [37,38], and the consideration of new datasets (for instance the Möller dataset [39]).

## Methods

### Introduction

Our approach considers the concatenation of the protein symbols with the inverted annotation string, the whole considered as an ELL string. We subsequently apply a transformation to it, in order to obtain a string belonging to a regular language. The transformation is done by joining the first symbol of the first half with the last of the second one, the second symbol of the first half with the second-last symbol, and so on. Then, a GI process learns a language building a transducer that accepts the first part of each symbol (the one coming from the first half of the string) and returns the second part as output. The test phase consists in using Viterbi's algorithm to analyse the string. This algorithm returns the transduction that is most likely to be produced by the input string.

### Notation and definitions

Let Σ be an alphabet and Σ* the set of words over the alphabet. A language is any subset of Σ*, that is a set of words. For any word *x *over Σ* let *x*_*i *_denote the *i*-th symbol of the sequence. Let |*x*| denote the length of the word and let *x*^*r *^denote the reverse of *x*. Let also *λ *denote the empty word. A grammar is denoted by *G *= (*N*, Σ, *P*, *S*) where *N *and Σ are the auxiliar and terminal alphabets, *P *is the set of productions and *S *∈ *N *is the initial symbol or axiom. Intuitively, a grammar can be seen as a rewritting system that uses the set of productions to generate a set of words over Σ*. The language generated by a grammar *G *is denoted by *L*(*G*).

An *Even Linear Grammar (ELG) *is a context-free grammar [40] where the productions are of the forms:

$$\begin{array}{ll} A\to xBy & \text{where }A,B\in N,x,y\in \Sigma^{*}\text{ and }|x|=|y|\\ A\to x & \text{where }A\in N,x\in \Sigma^{*} \end{array}$$

The class of Even linear Languages (ELL) is a subclass of the context free languages and includes properly the class of regular languages. Given an ELG, it is possible to obtain an equivalent one where the productions are of the form:

$$\begin{array}{ll} A\to aBb & \text{where }A,B\in N,a,b\in \Sigma \\ A\to a & \text{where }A\in N,a\in \Sigma \cup \{\lambda \} \end{array}$$

The learning of ELL can be reduced to the inference of regular languages [41]. The general algorithm consists in transforming the training strings through a function *σ*: Σ* → [Σ × Σ]* ∪ [Σ]* defined as follows:

$$\begin{array}{ll} \sigma (\lambda )=\lambda & \\ \sigma (a)=[a] & \text{where }a\in \Sigma \\ \sigma (axb)=[ab]\sigma (x) & \text{where }a,b\in \Sigma \text{ and }x\in \Sigma^{*} \end{array}$$

Intuitively, this function relates the first and last symbols of the word, as well as the second and the last but one, and so on. Once applied the function *σ*, it is possible to use any regular language inference algorithm to learn a language over the alphabet [Σ × Σ]* ∪ [Σ]*, that is, the alphabet of paired symbols. The learned language can be processed to undo the transformation *σ *as follows:

$$\begin{array}{l} \forall A\to [ab]B\in P\text{ add the production }A\to aBb\text{ to the ELG}\\ \forall A\to [a]\in P\text{ add the production }A\to a\text{ to the ELG}\\ \forall A\to \lambda \in P\text{ and all these productions to the ELG} \end{array}$$

Several inference algorithms are suitable to be applied, each obtaining a different solution. In fact, if the GI algorithm identifies a subclass of regular languages, then a subclass of ELL is obtained and applied with good performance.

**Figure 4:**
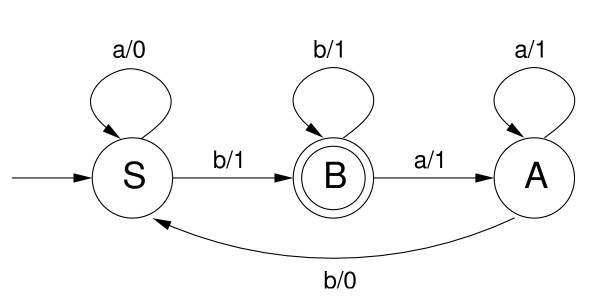
**A three states transducer example**. A label *x*/*y *denotes that the transition symbol is *x *with output *y*. For instance, the transduction of *baabaab *is 1110001

A *finite state transducer *is an abstract machine formally defined by a system *τ *= (*Q*, Σ, Δ, *q*_0_, *Q*_*F*_, *E*) where: *Q *is a set of states, Σ and Δ are respectively the input and output alphabets, *q*_0 _is the initial state, *Q*_*F *_⊆ *Q *is the set of final states and *E *⊆ (*Q *× Σ* × Δ* × *Q*) is the set of transitions of the transducer. A transducer processes an input string (word of a language), and outputs another string. A successful path in a transducer is a sequence of transitions (*q*_0_, *x*_1_, *y*_1_, *q*_1_), (*q*_1_, *x*_2_, *y*_2_, *q*_2_), ..., (*q*_*n*-1_, *x*_*n*_, *y*_*n*_, *q*_*n*_) where *q*_*n *_∈ *Q*_*F *_and for 1 ≤ *i *≤ *n*: *q*_*i *_∈ *Q*, *x*_*i *_∈ Σ* and *y*_*i *_∈ Δ*. Note that a path can be denoted as (*q*_0_, *x*_1_*x*_2 _... *x*_*n*_, *y*_1_*y*_2 _... *y*_*n*_, *q*_*n*_) whenever the sequence of states are not of particular concern. A transduction is defined as a function *t*: Σ* → Δ* where *t*(*x*) = *y *if and only if there exist a successful path (*q*_0_, *x*, *y*, *q*_*n*_). Figure 4 shows an example of transducer, and the transduction that an accepted sequence generates. We refer the interested reader to [42].

### Grammatical inference approach to transmembrane segments prediction

We consider the transmembrane segments prediction problem as a transduction problem. That is, given an amino acid sequence, the output of our system is a sequence with the same length which distinguishes between those amino acids within transmembrane segment and those that are not. In our work, we took into account the special features of our problem to propose a method based on inference of ELL.

First of all, we had to transform the available data to obtain a training set with even linear structure. This set was used to infer an ELL. The transducer is obtained using the structure of the inferred ELG. Given a ELG *G *= (*N*, Σ, *P*, *S*) that does not contain productions of the form *A *→ *a*, *a *∈ Σ, it is possible to obtain a transducer *τ *= (*N*, Σ, Σ, *S*, *Q*_*F*_, *E*) where:

$$\begin{array}{l} Q_{F}=\{A\in N:(A\to \lambda )\in P\}\\ E=\{(A,a,b,B):(A\to aBb)\in P\} \end{array}$$

Example 1 shows how this transformation work.

**Example 1 ***Given the ELG G *= (*N*, Σ, *P*, *S*) *with the productions:*

$$\begin{array}{l} S\to aS0|bB1\\ A\to aA1|bS0\\ B\to aA1|bB1|\lambda \end{array}$$

*then, the transducer τ *= (*N*, Σ, Σ, *S*, {*B*}, *E*) *is obtained where:*

$$E=\left\{\begin{array}{ccc} (S,a,0,S), & (S,b,1,B), & (A,a,1,A),\\ (A,b,0,S), & (B,a,1,A), & \left(B,b,1,B\right) \end{array}\right\}$$

*The resulting transducer is shown in Figure *4.

As we stated before, the learning problem for ELL can be reduced to the problem of learning regular languages. In our work, in order to learn the ELL, we use an algorithm to infer *k-testable in the strict sense *(*k*-TSS) languages [25-27]. The class of *ktss *languages is contained into the regular languages one; it is characterized by the set of segments of length *k *that appear in the words of the language.

Our approach considered a set of protein sequences *P *with known transmembrane domains and another set *L *of strings over an alphabet of labels Δ = {*i*, *o*, *M*}. For each sequence *x *in *P*, a labeled sequence *l*_*x *_is obtained. The labelling allows to distinguish the transmembrane segments from the non-transmembrane ones. That is, given the string *x *= *x*_1_*x*_2 _... *x*_*n *_∈ *P *and its corresponding labeled string *l*_*x*_, = *l*_1_*l*_2 _... *l*_*n *_∈ *L*, *l*_*i *_= *M *whenever *x*_*i *_correspond to a transmembrane segment, *l*_*i *_= *i*, when correspond to a inner segment, and *l*_*i *_= *o*, when correspond to a outer segment.

These sets were combined to obtain another set, named *M*, with the strings $xl_{x}^{r}$. Note that the strings in this set have an even linear structure and an even length. The set *M *was used to obtain a probabilistic transducer by ELL inference. The general method is summarized in Algorithm 1.

The returned transducer can be used to analyse problem sequences to obtain the corresponding transduction. It is possible that the transducer may result to be non-deterministic and the test sequences may not belong to the language accepted by the transducer. Therefore, an error-correcting parser (for instance Viterbi's algorithm) is necessary to analyze the test sequences. We employed a standard configuration of Viterbi's algorithm used when a GI approach is applied to pattern recognition tasks (i.e. [33]).

### Complexity

The igTM method is composed by two phases: inference and analysis. The first one consists in inferring an transducer from the sequences of the dataset.

The execution time of the GI algorithm used in this work is linear with the size of the dataset. The space requirements of this step is bounded by |Σ|^*k*^, where Σ is the alphabet of the samples and *k *is the parameter of the *k*-tss algorithm. Therefore, depending on the parameter used, the automaton obtained can be relatively big. The transformation of the automaton into a transducer is bounded by a polynomial of degree *k*.

The execution time of the second phase, the analysis one, is linear respect the size of the string to analyse. The space requirements are bounded by the size of the transducer and the analized string.

## Authors' contributions

PP wrote the software and carried out the experimentation. MC performed the search of web resources. All authors contributed to the study and interpretation of the results. The paper was written by PP and DL. DL conceived the research and supervised the whole process. All authors read and approved the final manuscript.
